# Data mobilisation for historical records of vascular plants in eastern Asia: V. L. Komarov’s expedition to Far-Eastern Russia, China and Korea from 1895 to 1897

**DOI:** 10.3897/BDJ.13.e143631

**Published:** 2025-01-30

**Authors:** Chin Sung Chang, Kae Sun Chang, Hui Kim

**Affiliations:** 1 Seoul National University, Seoul, Republic of Korea Seoul National University Seoul Republic of Korea; 2 Korea National Arboretum, Pochun, Republic of Korea Korea National Arboretum Pochun Republic of Korea; 3 Mokpo National University, Muan, Republic of Korea Mokpo National University Muan Republic of Korea

**Keywords:** georeferencing, botanical records, V.L. Komarov, primary occurrence data

## Abstract

**Background:**

Historical collections of herbaria and literature play a crucial role in documenting biodiversity information. The botanical biodiversity of northern Asia is significantly understudied compared to other regions of China and Japan. In particular, the biodiversity patterns in China's three north-eastern provinces, North Korea and the Russian Far East remain poorly understood, with substantial gaps when compared to the records of species distributions in Japan, South Korea and inland China. The Komarov data, orginally written in Russian, required extensive efforts to georeference the 130-year-old Chinese and North Korean place names to their modern equivalents and translate historical names to their current forms. This study aims to restore the Komarov data, including both specimen records and occurrence data, to assist the broader scientific and environmental community in recovering key biodiversity data from the past of northeast Asia. The impetus for this work was the need to assign geographic coordinates to plant specimens collected in the region and to V.L. Komarov's observations as primary occurrence data from 1895 to 1897.

**New information:**

In this study, we present historical occurrence data obtained from the north-eastern Asian plant expedition carried out from 1895 to 1897 by V.L. Komarov in Far-Eastern Russia, Heilongjiang, Jilin, the eastern region of Liaoning in China and the northern region of Korea. The occurrences were georeferenced to more than 350 sites in Russia, China and Korea. All occurrences were georeferenced and species names were cross-checked and taxonomically updated using our own Asian plant checklist. The dataset consists of 21,114 primary occurrence records, comprising 6,956 specimens and 14,158 observation records. The outcome clearly shows that such initiatives can reveal an unexpected amount of highly valuable biodiversity information for “data-poor” regions.

## Introduction

The uneven distribution of information on the occurrence of species is a key issue in biogeographical analysis as well as in the planning of conservation measures (Diniz Filho et al. 2023, Linder and Hardy 2005, Prendergast et al. 1993). For historical and linguistic reasons, biodiversity information. based on specimens and literature, is not easily accessible to researchers in East Asia, as in many other transboundary regions (Chang et al. 2021a, Lohonya et al. 2020, Nundloll et al. 2022). Consequently, it is difficult to plan for adequate conservation and sustainable use of the region’s flora (Zhang et al. 2012). Biodiversity information in northeast Asia is known to be of poor quality, with information such as collection dates, place names and collectors missing or written in the wrong data format (Chang et al. 2021b, Anderson et al. 2020, Chapman et al. 2020). A major problem is that biodiversity organisations in East Asia have failed to develop the structured and relational software needed to manage the natural history collections that underpin biodiversity (Chang et al. 2021b). Data quality requires the development of data integrity for database management and an ongoing training system for data managers to correct errors (Endresen et al. 2019, Faith et al. 2013). Datasets in East Asia are not suitable for generating essential biodiversity variables due to large geographical gaps and lack of long-term data, particularly in North Korea (Chang et al. 2021b, McCarthy et al. 2021). A variety of approaches are needed to efficiently improve data availability in the region, adopt open-science principles and help fill existing data gaps. Our major efforts to mobilise biodiversity data from V.L. Komarov's records in Far Eastern Russia, north-eastern China and North Korea have made primary species occurrence data available in a global network of data publishing/access platforms (Chang 2024, Chang and Chang 2003,Chang and Choi 2004). Georeferencing for this dataset was conducted using Komarov's flora, which provides descriptions and locality information derived from observations as well as physical specimens. While we have georeferenced some herbarium specimens from a couple of herbaria (e.g. A and TI), it is important to note that major georeferencing efforts were made, based on the text records in the Flora. The general approach was to chronologically compile all available records of Komarov's specimens and observations in this region. The aim of this work was to develop a historical biodiversity dataset in terms of fitness-for-use (Anderson et al. 2020).

## Project description

### Title

V.L. Komarov botanical records from eastern Asia from 1895 to 1897

### Personnel

The datasets were digitised by Hui Kim (data manager), Chin S. Chang was the resource creator and Kae Sun Chang and Chin-Sung Chang were the content providers. With the help of Brahms (Filer 2001), C.S. Chang identified taxonomic changes in Komarov's nomenclature and geo-referenced in the species occurrence data.

### Design description

The project aims to mobilise and publish the data about V.L. Komarov's collections deposited at LE and other herbaria and primary occurrence data cited in Flora Manshuriae (Komarov 1901, Komarov 1904, Komarov 1907, Komarov 1927a, Komarov 1927b, Komarov 1927c, Komarov 1927d, Komarov 1927e, Komarov 1927f, Komarov 1932a, Komarov 1932b, Komarov 1932c).

### Funding

This research was supported by Korea National Arboretum (Data-mining project).

## Sampling methods

### Sampling description

**Figure 1.**
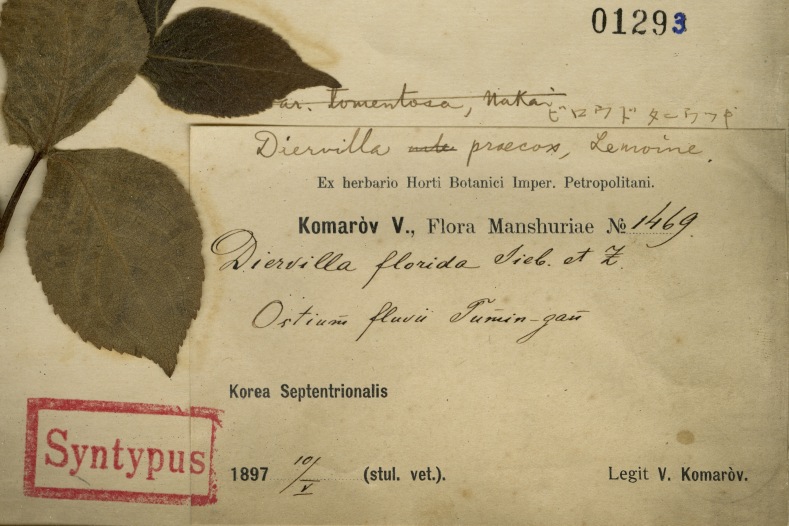
An example specimen label, Komarov1469, registered as *Diervillaflorida* (Bunge) Siebold & Zucc as well as the syntype of Weigelapraecoxvar.tomentosa Nakai. in the Herbarium of the University of Tokyo (TI), was collected from ‘*Ostium Fluvii Tumin-gan*’, a place name georeferenced to be located in Undok, Hamgyong-bukto, Democratic People's Republic of Korea. It was currently using the accepted name, *Weigelaflorida* (Bunge) A.DC.

**Figure 2.**
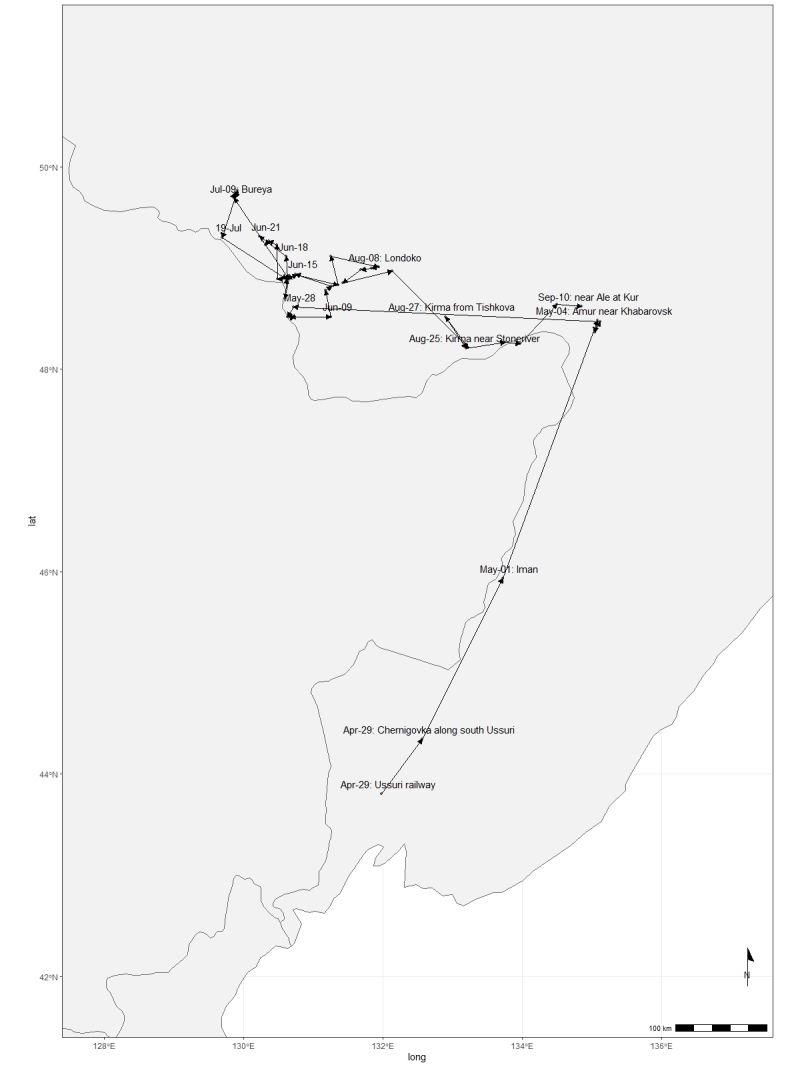
The map of the collecting area for the 1895 V.L. Komarov's botanical expedition. The route is not shown in detail on the map for the sake of clarity. However, the expedition actually started in Ussuri in April 1895. It ended in Pompeyevka in October 1895.

**Figure 3.**
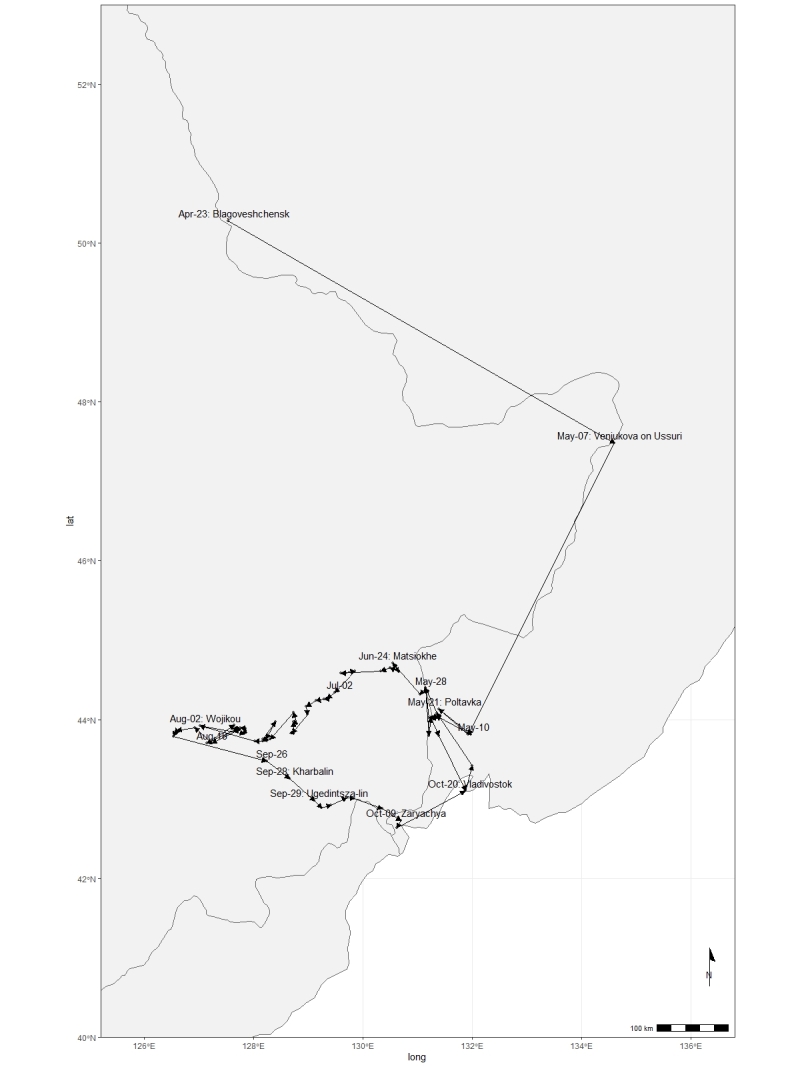
The map of the collecting area for the 1896 V.L. Komarov's botanical expedition. The route is not shown in detail on the map for the sake of clarity. However, the expedition actually started in Blagoveshchensk on the Amur in April 1896. It ended in Vladivostok in October 1896.

**Figure 4.**
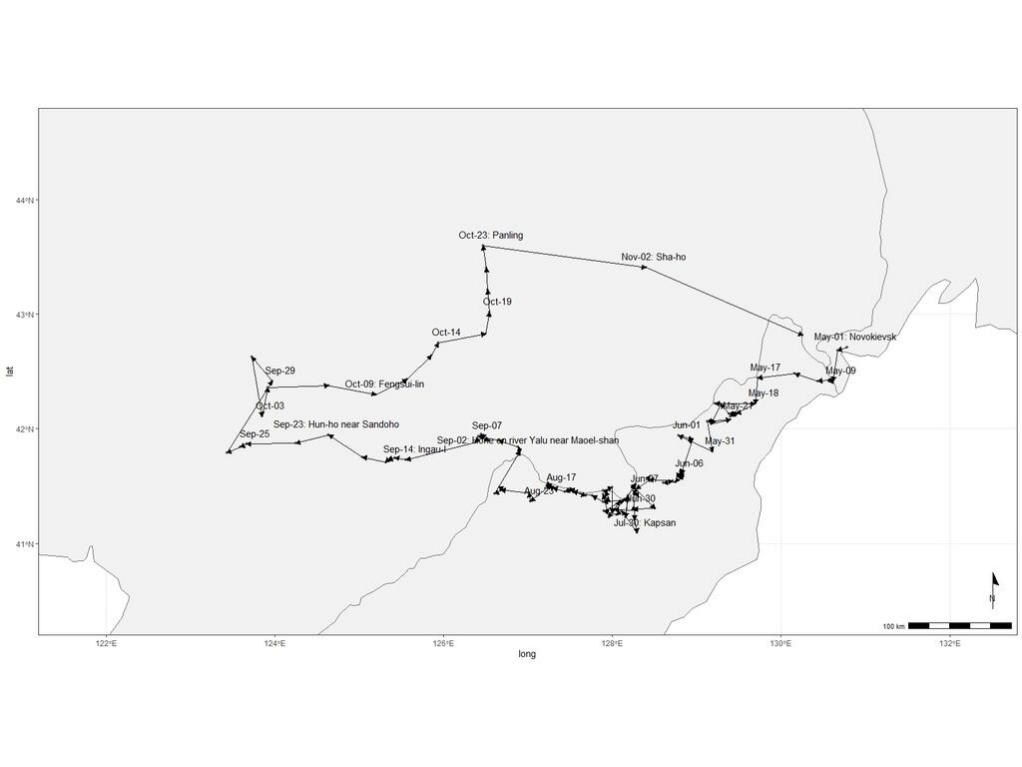
The map of the collecting area for the 1897 V.L. Komarov's botanical expedition. The route is not shown in detail on the map for the sake of clarity. However, the expedition actually started in Vladivostok in April 1897. It ended in Tumin-gan near Langsui-chuinda in November 1897.

Within the LE Herbarium, two groups have been defined for data mobilisation purposes. The first group consists of herbarium specimen records (Fig. 1) of vascular plants collected in the Far East of Russia, northeast China and North Korea by Komarov. V.L. Komarov collected 1,435 specimens in 1895, 1,834 specimens in 1896 and 3,687 specimens in 1897, along with plant records for 51 families that he identified. These data describe a specimen dataset of East Asian plants preserved in the Komarov Botanical Institute (LE) and duplicates distributed to several other institutes (like V, TI, NY, A, Stafleu and Cowan (1979)). The second group includes the primary occurrence data that he observed in the field. Komarov devoted three years to botanical research, meticulously documenting the species he observed in the field. In 1895, he documented 2,270 occurrences from the Amur and Ussuri Rivers in Russia (Fig. 2). The following year, 1896, he extended his observations, documenting another 3,872 occurrences from Primorsky and Amur in Russia and Jilin, Liaoning and Heilongjiang in China (Fig. 3). By 1897, Komarov's efforts had culminated in the recording of another 8,016 occurrences from Hamgyong-bukto, Ryanggang, Chagang-do in North Korea and Jilin, Liaoning in China (Fig. 4). The data from the Komarov's literature were transferred to the draft tables according to the GBIF requirements for occurrence datasets (Robertson et al. 2014).

### Quality control

Georeferencing: In the case of Komarov's geographic name information, the original Russian-language Flora Manshuriae (Komarov 1901, Komarov 1904, Komarov 1907) transcribed the locality names in Russian Cyrillic, while the later Japanese-translated version (Komarov 1927a, Komarov 1927b, Komarov 1927c, Komarov 1927d, Komarov 1927e, Komarov 1927f, Komarov 1932a, Komarov 1932b, Komarov 1932c) by the South Manchurian Railway Company presents the place names, based on Chinese characters. These two different scribal records, the historically recorded Chinese characters in northern China and on the Korean Peninsula and Komarov's early gazetteer with its corresponding transliterations, are the main sources of information for the georeferencing calculations in this dataset. In many Asian countries, Japanese exonyms are geographic names in the Japanese language that differ from those in the dominant language and these Japanese terms for some names are now a mystery, either because they are quite different from endonyms or because of some other obscure etymology, making it difficult to determine past collection sites (Chang et al. 2021a). Komarov's gazetteer existed before the Japanese altered the place names, and the Japanese version of the gazetteer helped rather than hindered our georeferencing because it relied on locally used Chinese characters rather than Japanese exonyms. Chang et al. (2015) has already published a multilingual botanical gazetteer, which was used in this study to resolve inconsistencies, uncertainties and confusion about the geographic names on the Korean Peninsula used by foreign explorers and Korean plant collectors over the past 120 years. Once the place names have been identified, the next step was to provide an accurate match to a biological collection. We always aimed for accurate georeferencing for place coordinates, but sometimes this was not possible due to insufficient information in the place names. In these situations, we used higher geographic area coordinates, such as counties or cities. To minimise errors, improve data consistency and maintain integrity throughout the georeferencing process, we modified a procedure used by the Chinese Type Collection Project (Lohonya et al. 2020).

### Step description

1. The data mobilisation phase includes the extraction of data from the three volumes of Komarov's Flora Mandshuiae, the filling of the initial datasets and the georeferencing. Quality control is carried out at all stages and includes a final cross-check of the data. In particular, the master files and initial datasets will be archived in the BRAHMS database. We used the taxon module provided by BRAHMS to manage scientific names and we used the verbatim scientific names given by Komarov to reflect the most recent nomenclature (POWO 2024), together with the regional checklist (Chang et al. 2020).

2. The botanical records are taken from the three volumes of Flora Manshuriae, the lists of available material (gazetteers and data) being synchronised and updated according to the latest taxonomy. When only literature was available for species occurrence records, the scientific names recorded in Komarov's flora were transcribed into the current scientific names, with typographical errors removed. The rank of the original scientific name and the corresponding taxonomic status were retained. Where specimens were available, the earliest scientific name recorded on the label was checked against literature and the earliest scientific name recorded was chosen as the default scientific name. For types of new taxa by Komarov as well as other authors, the scientific name of the corresponding type is given in the "typeStatus", for example, "Isosyntype of Acertschonoskiivar.rubripes Kom.". Where the accepted name differed, we presented it in a column called 'acceptedNameUsage'.

3. A multilingual gazetteer has been produced to resolve the confusion of place names in northeast China, Far Eastern Russia and North Korea. The gazetteer thesaurus was created as a tool to correlate the often radically different names given to a single place or feature and to provide geographic coordinates for each.

4. The geographical information on the labels of this period included not only the name of the place where the specimen was collected, but also other information, such as a topographic description of the river or its location on a particular mountain. To know the distribution of a plant species, it is first necessary to georeference the collections so that they can be plotted on a map.

## Geographic coverage

### Description

Komarov journeyed through Amur Oblast, Jewish Autonomous Oblast and Khabarovsk Krai regions of Russia in 1895. The following year, in 1896, he explored the Primorsky Krai, Heilongjiang and Jilin regions of China. Subsequently, in 1897, he revisited the Primorsky Krai in Russia. His travels continued to lead him to Rason, Hamgyongbuk-do, Ryangang and Chagang in North Korea, as well as Liaoning and Jilin in China.

### Coordinates

40.88 and 49.46 Latitude; 122.87 and 133.43 Longitude.

## Taxonomic coverage

### Description

The majority of specimen records and primary occurrence data belong to class Magnoliopsida (4,669 specimens + 12,228 occurrence data from literature) and Liliopsida (1,605 specimens +1,337), followed by Polypodiopsida (445 specimens + 442), Lycopodiopsida (54 specimens + 43) and Coniferophyta (183 specimens + 108). Our dataset represents 123 families (Fig. 6), of which 13.9% of the data belong to the monocot families Poaceae (3.3%), Cyperaceae (2.5%), Orchidaceae (1.8%), Asparagaceae (1.3%) and Liliaceae (1.2%). The dicot comprises 80.0% of the total, followed by families Asteraceae (11.5%), Rosaceae (7.3%) and Ranunculaceae (6.0%), respectively, followed by Fabaceae (4.2%), Lamiaceae (3.2%), Apiaceae (2.9%), Caryophyllaceae (2.5%), Campanulaceae (2.2%), Betulaceae (2.1%), Caprifoliaceae (2.0%) and Salicaceae (1.9%). It further includes 604 genera, with the significant ones being *Artemisia* (416), *Acer* (396), Carex (334), *Viola* (297), *Polygonum* (271), *Salix* (255), *Potentilla* (247), *Betula* (243), *Clematis* (229), *Aster* (218), *Lespedeza* (209), *Aconitum* (207), *Vicia* (198), Saussurea (195), *Thalictrum* (193), *Ulmus* (184), *Angelica* (183), *Galium* (181), *Adenophora* (178), *Euonymus* (172), *Geranium* (165), *Prunus* (165), *Lilium* (162), *Cacalia* (158), *Quercus* (154), *Corylus* (149), *Lonicera* (144), *Lactuca* (141), *Veronica* (139), *Stellaria* (135), *Sedum* (132), *Tilia* (132), *Spiraea* (131), *Populus* (130), *Patrinia* (125), *Rosa* (125), *Athyrium* (119), *Chrysosplenium* (119) and *Iris* (118).

## Temporal coverage

### Notes

April 1895 to November 1897.

## Usage licence

### Usage licence

Creative Commons Public Domain Waiver (CC-Zero)

### IP rights notes

This work is licensed under a Creative Commons Attribution (CC-BY) 4.0 License.

## Data resources

### Data package title

V. L. Komarov’s expedition to Russia, China and Korea from 1895 to 1897

### Resource link

https://www.gbif.org/dataset/8f4ec71e-ca9f-42d6-9b08-008325dc5389

### Alternative identifiers

http://ipt.eabcn.net:8080/ipt-2.4.2/resource?r=komarov_east_asia

### Number of data sets

1

### Data set 1.

#### Data set name

V. L. Komarov’s expedition to Russia, China and Korea from 1895 to 1897

#### Data format

Darwin Core Archive

#### Description

The total dataset contains some 6,956 plant specimen records and 14,158 occurrences recorded by V. L. Komarov in northeast Asia during the three-year period from 1895 to 1897. These data are expected to contribute to biodiversity management and conservation of ecosystems in these highly inaccessible regions.

**Data set 1.** 

Column label	Column description
institutionCode	The name (or acronym) in use by the institution having custody of the object(s) or information referred to in the record.
type	The nature or genre of the resource.
basisOfRecord	The specific nature of the data record.
occurrenceID	An identifier for the Occurrence (as opposed to a particular digital record of the occurrence). In the absence of a persistent global unique identifier, construct one from a combination of identifiers in the record that will most closely make the occurrenceID globally unique.
recordedBy	A list (concatenated and separated) of names of people, groups or organisations responsible for recording the original Occurrence. The primary collector or observer, especially the one who applies a personal identifier (recordNumber), should be listed first.
recordNumber	An identifier given to the Occurrence at the time it was recorded. Often serves as a link between field notes and an Occurrence record, such as a specimen collector's number.
typeStatus	A list (concatenated and separated) of nomenclatural types (type status, typified scientific name, publication) applied to the subject.
eventDate	The date-time or interval during which an Event occurred. For occurrences, this is the date-time when the event was recorded. Not suitable for a time in a geological context.
DAY	The integer day of the month on which the Event occurred.
MONTH	The integer month in which the Event occurred.
YEAR	The four-digit year in which the Event occurred, according to the Common Era Calendar.
taxonomicStatus	The status of the use of the scientificName as a label for a taxon. Requires taxonomic opinion to define the scope of a taxon. Rules of priority then are used to define the taxonomic status of the nomenclature contained in that scope, combined with the expert's opinion. It must be linked to a specific taxonomic reference that defines the concept.
country	The name of the country or major administrative unit in which the Location occurs.
countryCode	The standard code for the country in which the Location occurs.
stateProvince	The name of the next smaller administrative region than country (state, province, canton, department, region etc.) in which the Location occurs.
county	The full, unabbreviated name of the next smaller administrative region than stateProvince (county, shire, department etc.) in which the Location occurs.
locality	The specific description of the place. Less specific geographic information can be provided in other geographic terms (higherGeography, continent, country, stateProvince, county, municipality, waterBody, island, islandGroup). This term may contain information modified from the original to correct perceived errors or standardise the description.
decimalLatitude	The geographic latitude (in decimal degrees, using the spatial reference system given in geodeticDatum) of the geographic centre of a Location. Positive values are north of the Equator, negative values are south of it. Legal values lie between -90 and 90, inclusive.
decimalLongitude	The geographic longitude (in decimal degrees, using the spatial reference system given in geodeticDatum) of the geographic centre of a Location. Positive values are east of the Greenwich Meridian, negative values are west of it. Legal values lie between -180 and 180, inclusive.
geodeticDatum	The ellipsoid, geodetic datum or spatial reference system (SRS) upon which the geographic coordinates given in decimalLatitude and decimalLongitude are based.
minimumElevationInMetres	The lower limit of the range of elevation (altitude, usually above sea level), in metres.
vernacularName	A common or vernacular name.
GENUS	The full scientific name of the genus in which the taxon is classified.
specificEpithet	The name of the first or species epithet of the scientificName.
taxonRank	The taxonomic rank of the most specific name in the scientificName.
infraspecificEpithet	The name of the lowest or terminal infraspecific epithet of the scientificName, excluding any rank designation.
acceptedNameUsage	The full name, with authorship and date information, if known, of the currently accepted taxon.
scientificName	The full scientific name, with authorship and date information, if known. When forming part of an Identification, this should be the name in lowest level taxonomic rank that can be determined. This term should not contain identification qualifications, which should instead be supplied in the IdentificationQualifier term.
kingdom	The full scientific name of the kingdom in which the taxon is classified.
phylum	The full scientific name of the phylum or division in which the taxon is classified.
class	The full scientific name of the class in which the taxon is classified.
order	The full scientific name of the order in which the taxon is classified.
family	The full scientific name of the family in which the taxon is classified.

## Additional information

Biographical notes

**Figure 5.**
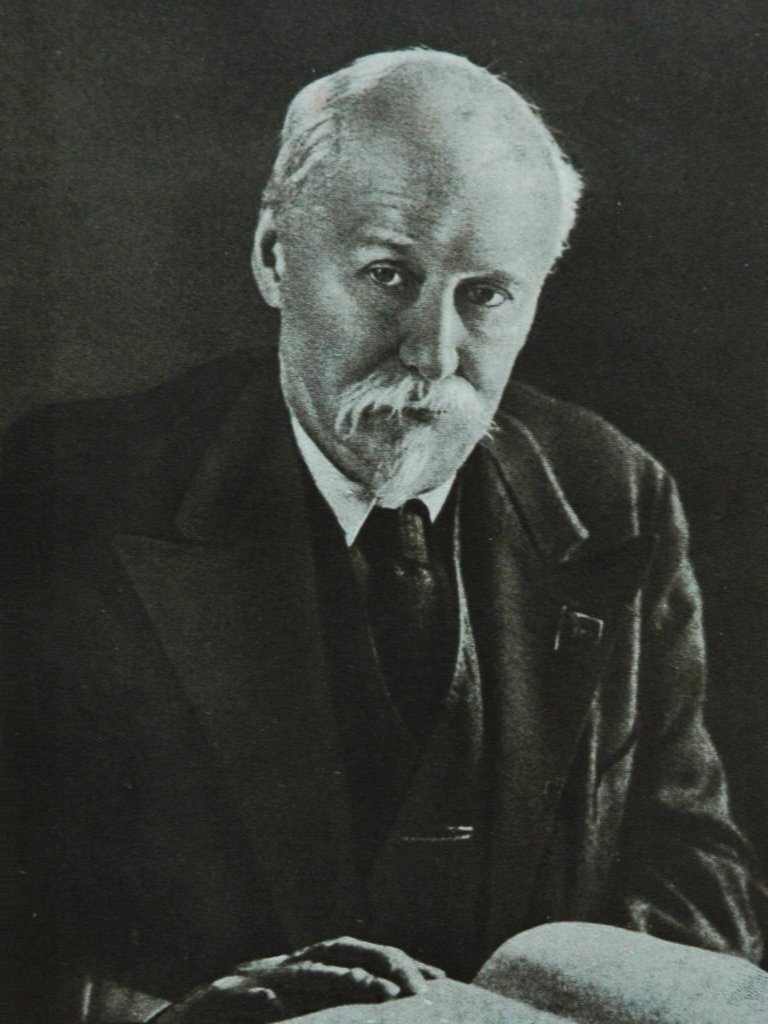
The portrait of V. L. Komarov (1869-1945) from Wikimedia common website.

**Figure 6.**
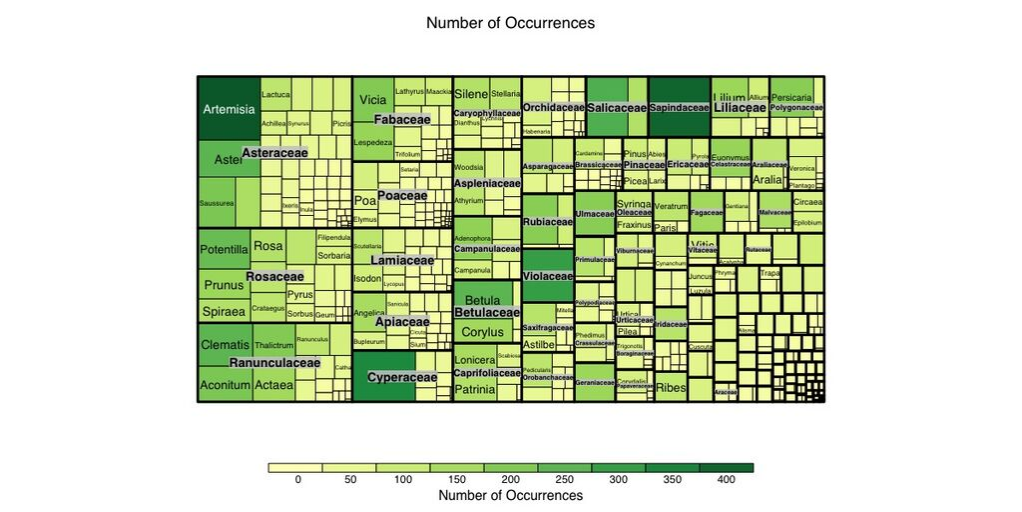
Taxonomic distribution of occurrences amongst vascular plant genera and families in the dataset. The figure was prepared with the “treemap” package in R (Tennekes 2010).

A Russian botanist, Vladimir Leontievitch Komarov (1869-1945) was born at St. Petersburg and graduated from St. Petersburg University in 1894 (Ashby 1946). When he was at the age of 26, Komarov obtained through the Imperial Russian Geographical Society permission for a three-year expedition to the Far East. In May 1895, he started his field expeditions to Amur River region of Far-Eastern Russia and continued his trip to Ussuri River region of Russia and eastern part of north-eastern China (called Manchuria; southern Heilongjiang and northern part of Jilin) in 1896 (Ilizarov 2021). Komarov’s exploration region of North Korea in 1897 lay at the eastern edge of Russia and at the northern limit of China and extended from the western part of Far-Eastern Russia to south of Jilin Province of China. Most of the region lay in what were once the Hamkyongbuk-do Provinces of Korea, which were divided into two provinces including newly-formed Ryanggang-do. V. L. Komarov was best known for his contribution to Amur and Ussuri flora of Far-Eastern Russia, north-eastern China flora and North Korea flora (Fig. 5).

### The significance of this dataset

**Figure 7.**
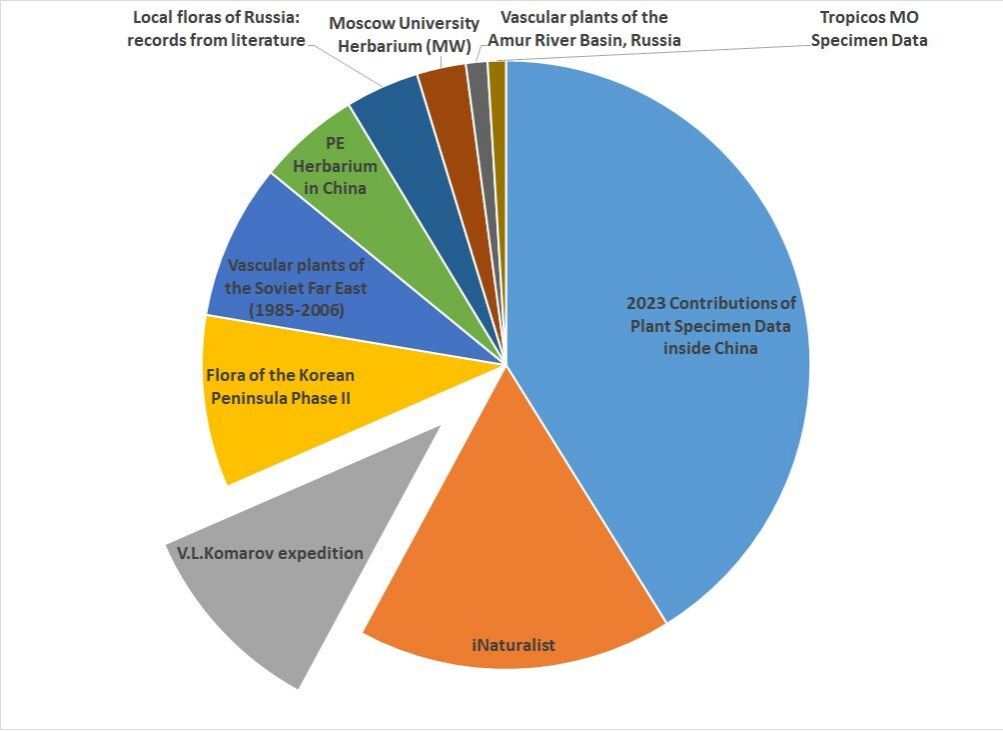
Pie chart for "2023 Contributions of Plant Specimen Data inside China" ranked first to "Tropicos MO Specimen Data" ranked tenth, in order of the number of vascular plant occurrences provided within the bounding box presented in the text.

A rectangular box (N 41.0928 - N 45.3540, E 122.8110 - E 132.0800) encompassing the two-year expedition between 1896 and 1897 was used to determine the contribution of species occurrence data from all vascular plant datasets in the region (Fig. 7). Out of a total of 164,064 occurrences, 17,353 (10.6%) were identified as originating from our dataset. It is the third largest dataset after '2023 Contributions of Plant Specimen Data inside China' and the iNaturalist data and adds a significant amount of data to a relatively under-represented region.
